# Innate immune function and antioxidant capacity of nestlings of an African raptor covary with the level of urbanisation around breeding territories

**DOI:** 10.1111/1365-2656.13837

**Published:** 2022-12-04

**Authors:** Chima Josiah Nwaogu, Arjun Amar, Carina Nebel, Caroline Isaksson, Arne Hegemann, Petra Sumasgutner

**Affiliations:** ^1^ FitzPatrick Institute of African Ornithology, DST‐NRF Centre of Excellence University of Cape Town Cape Town South Africa; ^2^ Department of Biology University of Turku Turku Finland; ^3^ Department of Biology Lund University Lund Sweden; ^4^ Konrad Lorenz Research Centre, Core Facility for Behaviour and Cognition University of Vienna Grünau/Almtal Austria; ^5^ Department of Behavioural & Cognitive Biology University of Vienna Vienna Austria

**Keywords:** early‐life environment, ecological immunology, environmental seasonality, landscape ecology, oxidative balance, path analysis, urban raptor

## Abstract

1. Urban areas provide breeding habitats for many species. However, animals raised in urban environments face challenges such as altered food availability and quality, pollution and pathogen assemblages. These challenges can affect physiological processes such as immune function and antioxidant defences which are important for fitness.
2. Here, we explore how levels of urbanisation influence innate immune function, immune response to a mimicked bacterial infection and antioxidant capacity of nestling Black Sparrowhawks *Accipiter melanoleucus* in South Africa*.* We also explore the effect of timing of breeding and rainfall on physiology since both can influence the environmental condition under which nestlings are raised. Finally, because urbanisation can influence immune function indirectly, we use path analyses to explore direct and indirect associations between urbanisation, immune function and oxidative stress.
3. We obtained measures of innate immunity (haptoglobin, lysis, agglutination, bactericidal capacity), indices of antioxidant capacity (total non‐enzymatic antioxidant capacity (tAOX) and total glutathione from nestlings from 2015 to 2019. In addition, in 2018 and 2019, we mimicked a bacterial infection by injecting nestlings with lipopolysaccharide and quantified their immune response.
4. Increased urban cover was associated with an increase in lysis and a decrease in tAOX, but not with any of the other physiological parameters. Furthermore, except for agglutination, no physiological parameters were associated with the timing of breeding. Lysis and bactericidal capacity, however, varied consistently with the annual rainfall pattern. Immune response to a mimicked a bacterial infection decreased with urban cover but not with the timing of breeding nor rainfall. Our path analyses suggested indirect associations between urban cover and some immune indices via tAOX but not via the timing of breeding.
5. Our results show that early‐life development in an urban environment is associated with variation in immune and antioxidant functions. The direct association between urbanisation and antioxidant capacity and their impact on immune function is likely an important factor mediating the impact of urbanisation on urban‐dwelling animals. Future studies should explore how these results are linked to fitness and whether the responses are adaptive for urban‐dwelling species.

## Graphical abstract

This study presents a new viewpoint from which the impact of urbanisation on wildlife can be considered, that is, through the impact of urbanisation on phenology and the associations between phenology, physiology and seasonal environmental change.

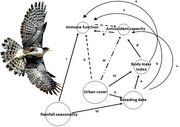

## 1 INTRODUCTION

Urban landscapes are rapidly expanding across the globe (McKinney, 2002). These landscapes are characterised by several potential stressors for urban‐dwelling wildlife. For example, they often have altered resource availability (Cox & Gaston, 2018), higher levels of pollution (Bauerová et al., 2017) and novel pathogen assemblages (Bradley & Altizer, 2007; Delgado‐V & French, 2012), all of which can affect individual health and ultimately fitness. Although some species thrive in urban environments, long‐term detrimental impacts of an urban life such as telomere shortening (Ibáñez‐Álamo et al., 2018; Salmón et al., 2016), an increased stress response (Beaugeard et al., 2019) and compromised innate immune system have been observed, especially if exposure to urban habitats and associated stressors occur during early‐life development (Salmón et al., 2017; Ziegler et al., 2021). To understand the mechanisms underlying emerging threats from increased urbanisation, it is important to investigate how animals adjust their physiological defences to urbanisation.

Urban‐dwelling animals are exposed to stressors that can increase susceptibility to disease (Bradley & Altizer, 2007) and this can be especially problematic for young animals, since their immune system is less developed (Aastrup & Hegemann, 2021; Arriero et al., 2013; Stambaugh et al., 2011). Hence, they may have a reduced ability to deal with immune challenges. In birds, nestlings may have an under developed antibody response which may persist until fledging for altricial species (Killpack et al., 2013; Killpack & Karasov, 2012; Lee, 2006) and this may increase their susceptibility to disease upon exposure to infection. In addition to other processes, maternal antibodies play a vital role in protecting young animals from infection during this period of seemingly weak immune defence (Fortuna et al., 2021; Grindstaff, 2008; Hasselquist & Nilsson, 2009; Pihlaja et al., 2006). Urban habitats that expose young animal to high infection risk can act as ecological traps for urban breeding animals (Demeyrier et al., 2016; Schlaepfer et al., 2002; Stracey & Robinson, 2012) which may exploit the seemingly suitable breeding conditions (such as high food availability and low predation risk) in urban habitats, but then are unable to successfully raise offspring that will survive to breed themselves. Constitutive innate immune function can reduce disease susceptibility and the strength of the innate immune function should match the risk of infection (Horrocks et al., 2011), allowing animals to avoid diseases and costly inflammatory responses that can invoke oxidative stress during infection (Hasselquist & Nilsson, 2012).

**FIGURE 1:**
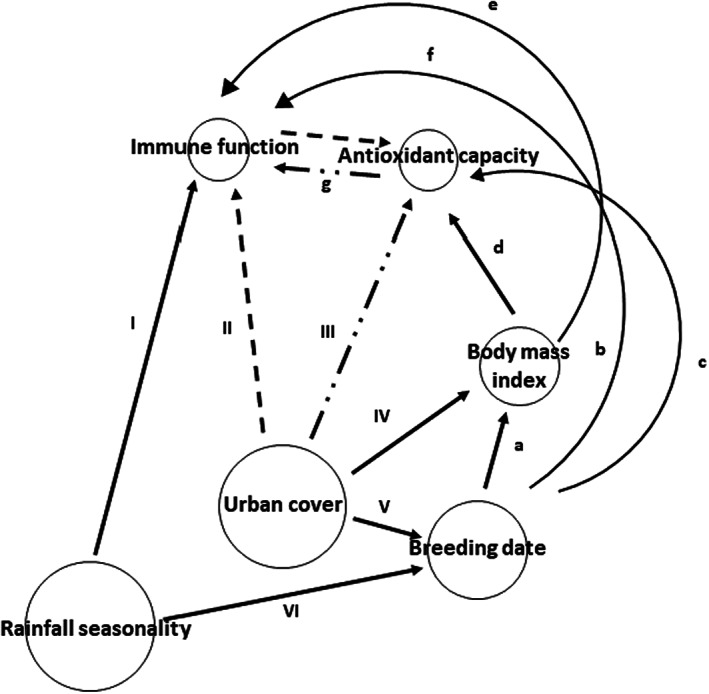
Urbanisation (and other environmental factors like rainfall) may affect immune function, antioxidant capacity, body mass index and breeding date directly (I, II, III, IV, V & VI) or indirectly via other factors (a, b, c, d, e, f & g). Immune indices and antioxidant capacity may covary with urbanisation (g) because urbanisation can affect both factors independently or through one of immune function or antioxidant capacity: Dashed lines—urbanisation affects immune function and immune function affects antioxidant capacity, or alternatively, dot‐dashed lines—Urbanisation affects antioxidant capacity and antioxidant capacity affects immune function. Other indirect effects of urbanisation may arise from its direct or indirect effect on breeding date and body mass index.

Upregulation of immune function in urban environments can cause oxidative stress (Costantini & Møller, 2009), but animals in urban environments may experience oxidative stress that is unrelated to immune responses (Isaksson, 2015). Urbanisation is often associated with changes in antioxidant capacity because antioxidants can be depleted by poor diet (Isaksson, 2018), pollution (Bauerová et al., 2017) and infection (Toomey et al., 2010). Therefore, immune function and antioxidant capacity may be connected directly or indirectly through urbanisation or factors affected by urbanisation (Figure 1). Comprehensive studies investigating the occurrence and determinants of these relationships are generally lacking (but see Ibáñez‐Álamo et al., 2020). Moreover, in contrast to temperate regions (Bonier, 2012; Partecke et al., 2006; Sepp et al., 2019), there are far fewer studies investigating relationships between urbanisation and defence physiology in tropical and sub‐tropical regions. Yet, these regions are characterised by expanding urban landscapes (Güneralp et al., 2017; Sumasgutner, 2021), altered resource availability with implications for individual body condition (Cox & Gaston, 2018; Meillère et al., 2015) and by higher infection rates (Altizer et al., 2006, 2013; Harvell et al., 2009). Studies covering different physiological processes from different regions are needed to better understand the impact of urbanisation on wildlife. Ideally, such studies should incorporate other potential influences on physiology such as spatiotemporal environmental variables such as rainfall (Ndithia et al., 2017, 2019; Nwaogu, Cresswell, & Tieleman, 2020; Tieleman et al., 2019), diet (Nwaogu, Galema, et al., 2020; Schultz et al., 2017) and individual variables like body mass (Hegemann et al., 2012) and should use robust analytical approaches capable of unravelling inter‐connectivity between potentially influential factors (Figure 1).

The complexity of the immune system entails that several immune indices that reflect different attributes of the immune system are required to comprehensively interpret variation in innate immune function (Adamo, 2004) under specific contexts (Boughton et al., 2011). Immune indices such as natural antibodies and complement activity or bactericidal capacity of whole blood or plasma reflect the potential to clear infection (Matson et al., 2005; Millet et al., 2007; Tieleman et al., 2005). Alternatively, haptoglobin is a biomarker of an ongoing inflammatory response which circulates in low concentration and increases in response to inflammation (Jain et al., 2011; Matson et al., 2012; Quaye, 2008; but see Hegemann et al., 2013). Thus, changes to haptoglobin concentration following an infection may reflect the intensity of immune response or the magnitude of an immune challenge. Similarly, antioxidant capacity is upregulated to prevent the self‐destruction that may arise from elevated levels of reactive oxygen species (ROS). Several indices may be required to infer antioxidant capacity. Indices such as total non‐enzymatic antioxidant capacity and total glutathione (tGSH) are considered useful and complementary measures of antioxidant capacity (Isaksson et al., 2011). Total non‐enzymatic antioxidant capacity is a plasma‐based index of antioxidant capacity which is affected by the systemic physiological and nutritional state, while tGSH is an endogenously synthesised antioxidant, often considered to be the most important intracellular antioxidant (e.g. Isaksson et al., 2011). The ratio of glutathione to its oxidised form, glutathione disulphide (recycled back to glutathione in a redox cycle), is a useful indicator of oxidative stress in cells and tissues, which can be very insightful when interpreted alongside total non‐enzymatic antioxidant capacity (Isaksson et al., 2011).

In this study, we adopt an integrated approach, using multiple measures of innate immunity and antioxidant capacity and quantified the immune response to a mimicked bacterial infection to explore the relationship between immune function and urbanisation, using nestling Black Sparrowhawks *Accipiter melanoleucus* raised on the Cape Peninsula, South Africa. In this area, Black Sparrowhawks have bred in territories across an extensive urban gradient since the first breeding record in 1993 (Oettlé, 1994). They colonised the Cape Peninsula through a south‐eastward expansion of their historic breeding range in South Africa (Martin, Koeslag, et al., 2014) and have simultaneously prolonged their breeding season to 10 months, lasting from March to November (Martin, Sebele, et al., 2014). Previous research found no negative impact of urbanisation on breeding or other population parameters (Rose et al., 2017; Sumasgutner et al., 2019; Suri et al., 2017). However, there is an indication that pairs in territories with greater urban cover breed earlier (Rose et al., 2017). Early breeders produce more offspring (Tate et al., 2017) which are more likely to be recruited into the breeding population (Sumasgutner, Tate, et al., 2016a). So, the impact of urbanisation may be confounded with the timing of breeding because pairs breeding in more urbanised territories (and thus, early) may differ in quality. Moreover, on the Cape Peninsula, temporal shift in breeding frequency corresponds with a unimodal variation in rainfall levels. Rainfall peaks around the middle of the breeding season (May to September; Cowling et al., 1996), so some nestlings are exposed to drier environmental conditions at the start or the end, compared with the middle of the breeding season. Such variation in environmental condition associated with the timing of breeding can help unravel how defence physiology responds to different environmental factors.

Using this study system, we explore how innate immune indices, antioxidant capacity and an immune response to a mimicked bacterial infection of nestling Black Sparrowhawks vary with urban cover, rainfall and timing of breeding. Using a path analysis, we then explore indirect associations between urban cover and immune function via timing of breeding, nestling body mass index and antioxidant capacity. We expect infection rates, and thus, immune function to be higher in more urbanised territories. Likewise, wetter periods of the breeding season should elicit elevated immune responses due to potentially higher infection rates influenced by higher environmental productivity (Tieleman et al., 2019). Similarly, antioxidant levels should increase in more urban territories to counter the negative consequences of high infection rates, pollution and other stressors that can enhance the internal pro‐oxidant levels if infection rates are higher in more urban territories. Over the breeding season, immune function may vary if the prevalence of infection, the physical condition of nestlings or their susceptibility to infection changes and cause them to upregulate or downregulate immune function. In rain‐driven seasonal environments, like on the Cape Peninsula, immune function and infection rates differ between seasons (Filion et al., 2020; Nwaogu et al., 2019). Additionally, research on Black Sparrowhawks on the Cape Peninsula so far suggests that infection of nestlings by *Leucocytozoon toddi* declines with increasing urban cover (Suri et al., 2017) and *Knemidokoptes* mite infection in adults is not associated with urbanisation or weather other pathogens may vary differently with environmental conditions (van Velden et al., 2017). More generally, bacteria, viruses and fungi are more abundant in rainy conditions due to high moisture and low ultraviolet radiation (Thomas et al., 2012). Arthropod vector‐borne pathogens also increase with higher vector abundance in the wet season (Berger et al., 2014) and novel pathogen assemblages are often associated with urban habitats (Bradley & Altizer, 2007; Hassell et al., 2017, 2019).

## 2 MATERIALS AND METHODS

### 2.1 Study system

In our study area on the Cape Peninsula, South Africa, Black Sparrowhawks breed in a matrix of habitat types including, sub‐urban gardens and woodlands comprising of mostly alien pine (e.g. *Pinus canariensis, Pinus elliotti, Pinus, halepensis, Pinus taeda* and *Pinus radiata*) and eucalyptus (*Eucalyptus tereticornis*). The climate is Mediterranean, experiencing winter rainfall between May and September (*c*. 400 mm/year), with peaks in June and July (Cowling et al., 1996). Breeding territories are maintained between seasons, although the nesting tree may change (Sumasgutner, Millán, et al., 2016).

For this study, we adopted the urban cover quantification used by Rose et al. (2017) and Sumasgutner et al. (2018), which estimates the percentage of urban cover (sealed and unproductive land areas) within a 2000 m radius surrounding each nest. This radius was derived from GPS tracking of adult male Black Sparrowhawks (Sumasgutner, Tate, et al., 2016c). Urban land cover was based on the South African National land‐cover dataset for land cover classes for 2013–2014 produced by GEOTERRA (Department of Environmental Affairs, 2015). This comprises 72 land cover types within each 30 m^2^ land area. From these, sealed and unproductive land areas were classified as ‘urban’, excluding coastal oceanic and open ocean areas. See Table S1 for extent of urban cover per territory and number chicks sampled per territory per year over the study period.

We expressed breeding date as the number of the days between the first of March (earliest month with egg‐laying record) and the date a nestling was sampled. We then calculated an index for rainfall variability for the entire study period (hereafter, ‘rainfall’), as the running total of daily rainfall 30 days prior (supplementary Figure S1)—total rainfall over the last 30 days. Rainfall data were obtained from the South African Weather Service weather station located at the Cape Town Airport (33.96, 18.60, altitude: 42 m).

### 2.2 Ethics statement

This study was conducted under CapeNature (Permit no. 0056‐AAA041‐00099, 0056‐AAA007‐00105, CN44‐30‐4175) and SanPark (Permit no. CRC/2015/009—2012, CRC/2017–2018/009—2012/V2) permits and approved by the UCT's ethics committee SFAEC (Permit numbers: 2012/v37/AA, 2016/v11/AA, 2018/v5/AA).

### 2.3 Field procedure and blood sampling

Black Sparrowhawks have been monitored on the Cape Peninsula since 2000. From March to November annually, territories with known breeding pairs of Black Sparrowhawks were visited monthly until breeding activity (i.e. courtship, mating, nest building or incubation) was observed after which weekly visits occurred. Nest checks were carried out from a vantage point away from the nest to avoid agitating the parents. Where it was not possible to view nestlings from the ground, we used a mounted mirror above the nest, where available, otherwise, we extrapolated nestling age from the perching behaviour of the brooding female. The perch pattern of the incubating female is often suggestive of the age of the nestlings. Nestlings were ringed at 20–35 (mean 28) days after hatching—these estimates of nestling age were verified during ringing by comparing their feather development with reference photos of nestlings with known age (Katzenberger et al., 2015).

Nestlings were sexed based on size (with males being *c*. 30% smaller than female based on tarsus length and body mass). We measured body mass (to nearest 1 g) and tarsus length (to nearest 0.1 mm). These measurements were used to calculate a body mass index, that is, the residuals of the linear regression between body mass and tarsus length controlling for sex (see Tate et al., 2016). From 2015 to 2019, we sampled blood from nestlings to measure innate immune function and antioxidant capacity. Blood samples were taken within 20 min of removing nestlings from the nest. This was within the time when no handling effect is expected on the nestling's immune function (Buehler et al., 2008; Zylberberg, 2015). About 0.2–0.5 ml of blood was collected from the brachial vein of each nestling using a heparinised syringe and needle. After sampling, we immediately placed a drop of whole blood (~10 μl) in an Eppendorf tube and stored it in liquid nitrogen for quantifying the tGSH concentration (see below). The remaining whole blood was centrifuged at 10,000 rpm/20 min to separate red blood cells from plasma, and both were stored in liquid nitrogen in the field. Samples were then stored at −80°C until assays were carried out.

In 2018 and 2019, we challenged nestlings with lipopolysaccharide (LPS)—a mimicked bacterial infection commonly used to assess immune responses (Hegemann et al., 2018; Matson et al., 2012; van de Crommenacker et al., 2010). We administered this immune challenge after sampling blood for innate immune indices and antioxidant capacity and measuring body morphometrics. We injected nestlings with 1 mg/kg body mass LPS (in phosphate‐buffered saline [PBS]) subcutaneously above the breast muscle. We injected all nestlings in the evening (between 15:00 and 17:33 h) and returned them to their nest for the night. Nestlings were removed from their nest the next morning (between 08:26 and 11:11 depending on time of LPS injection), sampled again and returned to their nest. To minimise stress, we did not resample nestlings that were not administered LPS injection. However, see Figure 1 for within‐individual increase in haptoglobin concentration following LPS‐injection and overall higher haptoglobin concentration in challenged versus unchallenged nestlings, supporting the assumption that this widely used immune challenge technique worked in this species.

### 2.4 Immune and antioxidant capacity assays

We performed physiological assays in four batches that correspond to specific sampling years: 2015 (batch 1), 2016 and 2017 (batch 2), 2018 (batch 3) and 2019 (batch 4). For each batch, samples were randomised before laboratory analyses.

#### 2.4.1 Agglutination/lysis titres

We assessed natural antibody‐mediated agglutination and complement‐mediated lysis of 1% rabbit red blood cells (Envigo RMS [UK] Ltd) in a serial dilution of 20 μl of Black Sparrowhawk plasma, following the methods described by Matson et al. (2005). We included duplicate chicken *Gallus gallus domesticus* plasma samples per plate as controls. We scored agglutination and lysis blind to sample identity using an existing protocol (Matson et al., 2005). Each sample was scored twice. If sample scores differed by more than one unit, we scored them a third time. For analysis, we used the mean (of two scores) or median (of three scores). The inter‐plate coefficient of variation (CV) of the chicken plasma for agglutination and lysis were 16.3% and 10.6%, respectively.

#### 2.4.2 Pre‐challenge haptoglobin concentration

We quantified haptoglobin concentration in 7.5 μl of plasma, using a functional colorimetric assay which quantifies haem‐binding capacity. We followed instructions for the ‘manual method’ provided with a commercially available assay kit (Cat. No.: TP801; Tridelta Development Ltd, Maynooth, Co.; Matson et al., 2012). A five‐step serial dilution (2.500, 1.250, 0.625, 0.312, 0.156 and 0.078 mg/mL) of haptoglobin standard was used as the standard curve concentrations. As a control, we added the manufacturer's pool on each plate (inter‐plate CV: 5.4%). In addition, we added a blank to each plate which was used to correct the haptoglobin concentrations of samples, pool and standard curves. We calculated haptoglobin concentration from a final absorbance reading taken at 650 nm. We took an additional absorbance reading at 450 nm before adding the final reagent. We included this reading as a covariate in our models where it was significant, to control for variation in plasma redness by absorbing the variation in haptoglobin concentration explained by differences in plasma redness.

#### 2.4.3 Bacteria killing assay

We quantified the bactericidal capacity of plasma against *Escherichia coli*, following the method described by French and Neuman‐Lee (2012) with a few modifications following Eikenaar and Hegemann (2016) (i.e. we reduced all reagents by one‐third and measured final absorbance at 600 nm wavelength): we mixed 4.5 μl of plasma, 3.5 μl of 10^5^ *E. coli*/ml and 8 μl PBS solution in microplates and ran all samples in triplicates. Each plate contained a positive control (only containing *E. coli* and no plasma) and a negative control (only containing PBS) in quadruplicates. We read absorbance at 600 nm, and this we considered as the background absorbance. Then, we incubated the plate at 37°C for 12 h and read the final absorbance at 600 nm. To obtain the bactericidal capacity, we first subtracted background absorbance from the final absorbance readings and calculated the difference (in %) between mean of positive controls and mean of plasma sample. The intra‐assay coefficient for the bacteria‐killing assay was 7.9%.

#### 2.4.4 Total non‐enzymatic antioxidant capacity and uric acid

To obtain the total non‐enzymatic antioxidant capacity (tAOX), we performed the ferric reducing antioxidant power (FRAP) assay (Benzie & Strain, 1996) which measures the overall ferric reducing capacity. Briefly, 5 μl of plasma was diluted 1:8 with ddH_2_O, then 20 μl of the diluted plasma sample was incubated with 150 μl working solution for 20 min at room temperature. Working solution contain sodium acetate trihydrate +2, 4, 6‐Tris (2‐pyridyl)‐s‐triazine (TPTZ) + iron (III) chloride hexahydrate (FeCl_3_–6H_2_O) in the ratio 10:1:1. Immediately after incubation, we measured the colour generated from the reduction of Fe^3+^ (ferric) to Fe^2+^ (ferrous) using a microplate reader (FLUOstar Omega, BMG Labtech) at 593 nm. The sample concentration was calculated using a standard curve of Fe^2+^ (iron (II) sulphate heptahydrate (FeSO_4_–7H_2_O), ranging between 0.094 and 3 mM). In addition, uric acid was measured because it is known to influence the FRAP assay (Eikenaar et al., 2017). Although uric acid has important antioxidant properties, the variation caused by uric acid is mainly driven by purine metabolism, which are high in carnivores. Uric acid concentration was measured from 5 μl of plasma using a commercial kit from SPINREACT (Sant Esteve de Bas), following the manufacturer's instructions.

The plasma samples for both FRAP and uric acid assays were run in duplicates and only samples with a CV of less than 10% were included. Each plate contained an inter‐assay control consisting of a pool of several Black Sparrowhawk plasma samples. The FRAP and uric acid concentration were positively correlated (*F*_1,220_ = 380.97, *p* < 0.001, *R* ^2^ = 0.63). Therefore, to obtain the tAOX, we extracted the residuals of a linear regression of FRAP against uric acid (Cram et al., 2015; Kilgas et al., 2010).

#### 2.4.5 Total glutathione

We determined the tGSH (both the reduced and oxidised form, tGSH) content of whole blood following Baker et al. (1990) and Isaksson (2013), with minor modifications. Briefly, 16 μl of 5% sulphosalicylic acid (SSA) to concentrations was added to 4 μl of whole blood to lyse the cells. After centrifugation, 10 μl of the supernatant, containing tGSH, was diluted with 200 μl GSH buffer (143 mM NaH_2_PO_4_, 6.3 mM EDTA, pH 7.4). Next, 20 μl of diluted samples and GSH standards were transferred into respective wells, followed by 200 μl of room‐tempered reaction solution (10 mM DTNB (10%), 2 mM NADPH (17%) and GSH buffer (73%)). The plate was placed in a plate reader and 5 μl of glutathione reductase (0.34 units/sample) was added and shaken to convert all oxidised GSH to its reduced form, performed automatically by the microplate reader (FLUOstar Omega, BMG Labtech). The absorbance was read at 412 nm at 30 s interval for 5 min (kinetic mode). We ran samples in duplicates and every 96‐well plate included a blank and an inter‐assay standard consisting of a pool of several Black Sparrowhawk whole blood samples. tGSH concentrations were calculated based on tGSH concentrations in the standard curve (3.12 μM‐10 μM GSH dissolved in 0.6% SSA).

#### 2.4.6 Response haptoglobin concentration (post‐challenge)

A change in haptoglobin concentration following an infection reflects the intensity of immune response or the magnitude of an immune challenge (Matson et al., 2012). Thus, we measured haptoglobin concentration (methods described above) from blood plasma samples collected post‐LPS injection and estimated immune response as the difference between haptoglobin concentration before and after LPS injection.

### 2.5 Statistical analyses

#### 2.5.1 Impact of urban cover, breeding date and rainfall on innate immune function and antioxidants

We used linear mixed models to explore how immune indices and antioxidants vary with urban cover, breeding date or rainfall. We built separate models with urban cover or breeding date as explanatory variables due to collinearity between these variables (Table S2). For each model, in addition to urban cover or breeding date, rainfall, body mass index, nestling age and sex were included as explanatory variables and two‐way interactions between nestling age and urban cover or breeding date. Nestling age and rainfall were retained in all our models whether their predictive power was significant or not to account for differences in nestling age and rainfall at the time of sampling. To account for inter‐annual variability in environmental condition and any differences that might arise from sample handling between years (e.g. reagents batch, transport, and storage time), year was included as a random factor. Territory was also included as a random term to account for variation arising from relatedness or territory effects. See Table 1 for repeatability estimates showing the proportion of variance explained by year and territory for all immune indices and antioxidants. Values of haptoglobin concentration were log‐transformed to improve normality. For all models, we implemented a backward elimination of non‐significant explanatory variables—while retaining age, urban cover, breeding date or rainfall—to arrive at the best predictor(s) of variation in immune indices and antioxidant capacity. We report the summary statistics for each variable at the time of its elimination from the model.

**TABLE 1:** Proportion of variation in immune function and antioxidant capacity indices of nestling Black Sparrowhawks explained by territory and year shown by repeatability estimates. Summarised repeatability estimates for territory and year. N: Samples size used for analyses—number of territories or years/number of samples. R: repeatability estimate (significant repeatability estimates are highlighted bold). CI: upper and lower confidence interval of repeatability estimates. tAOX: non‐enzymatic antioxidant capacity and tGSH: Total glutathione

Group	Lysis			Agglutination			Haptoglobin conc.			Bactericidal capacity			tAOX			tGSH		
	N	R	CI	N	R	CI	N	R	CI	N	R	CI	N	R	CI	N	R	CI
Territory	41/200	0.02	0–0.12	41/200	0.06	0–0.16	41/193	0.01	0–0.11	40/187	0.11*	0–0.34	41/195	0	0–0.03	39/169	0.03	0–0.16
Year	5/200	0.13*	0–0.33	5/200	0.09*	0–0.25	5/193	0.13*	0–0.31	5/187	0.11*	0–0.27	5/195	0.75*	0.24–0.90	5/169	0.04	0–0.15

#### 2.5.2 Impact of urban cover, breeding date and rainfall on immune response to a mimicked bacterial infection

**FIGURE 2:**
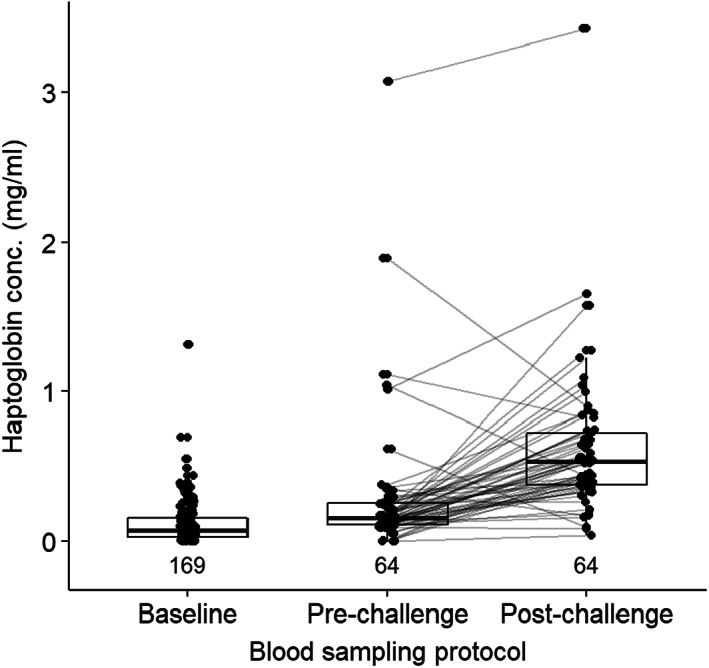
Haptoglobin concentrations of nestling Black Sparrowhawks in Cape Town, South Africa. baseline: Haptoglobin concentrations measured for individuals that were not immune‐challenged (years 2015–2019); pre‐challenge: Haptoglobin concentrations measured before the immune system were challenged via lipopolysaccharide injection; post‐challenge: Haptoglobin concentration measured ca. 17 h after the immune challenge was administered (years 2018 and 2019). The difference in haptoglobin concentration between blood plasma samples collected pre‐ and post‐challenge was used as an estimate of the immune response. Numbers in the figure show sample sizes. Lines connect points of the same individual and hence indicate within‐individual changes.

To explore whether a nestling's immune response to a mimicked infection varied according to urban cover and/or breeding date, we built general linear models with the difference between pre‐ and post‐challenge haptoglobin concentration (Figure 2) as the response variable. We again fitted separate models for urban cover or breeding date as explanatory variables. Additionally, in these models we also included rainfall, body mass index, nestling age and nestling sex. We tested interactions between nestling age and urban cover or breeding date, respectively, but as before, we retained rainfall and nestling age in our models regardless of their explanatory power. We transformed the immune response to account for negative differences between pre‐ and post‐challenge haptoglobin concentration and to improve normality: we added the difference between one and the minimum immune response to each value so that the resulting minimum immune response remained zero after log transformation. To test whether haptoglobin concentration created a ceiling in the ability of nestlings to respond to the immune challenge, we included the pre‐experiment haptoglobin concentration as an explanatory variable in all our models exploring change in haptoglobin concentration as a proxy for immune response (Hegemann et al., 2013). A ceiling in the ability of nestlings to respond would be indicated by a negative correlation between the pre‐ and the post‐challenge haptoglobin concentrations. Again, we implemented a backward elimination of non‐significant explanatory variables—while retaining age, urban cover, breeding date or rainfall—to arrive at the best predictor(s) of the immune response.

#### 2.5.3 Hypothetical indirect association between urban cover and immune function

We performed path analyses to explore whether any impact of urban cover on innate immune function arises indirectly via the timing of breeding, body mass and antioxidant capacity, or alternatively, whether any impact of urbanisation on antioxidant capacity was modulated via immune function, using the piecewisesem package in R (Lefcheck, 2015). Path analyses generate hypotheses about causal associations from correlative datasets (Shipley, 2009). Only tAOX was used for the path analyses because tGSH did not correlate with any of the immune indices we measured (Table S2). For each of lysis, agglutination, haptoglobin concentration and bactericidal capacity, we built a structural equation model, which tested the hypothetical relationship: urban cover predicts timing of breeding, timing of breeding predicts body mass index, body mass index predicts tAOX and tAOX predicts immune function. In addition, we tested whether urban cover predicted breeding date, body mass index or antioxidant capacity directly by including urban cover in all linear models. We included year and territory as random terms to account for inter‐annual variability and variation explained by nest and territory effects. The process was repeated for the alternative hypothesis: urban cover predicts timing of breeding, timing of breeding predicts body mass index, body mass index predicts immune function, and immune function predicts tAOX and the AICs of the alternative structural equation models were compared.

All statistical analyses were implemented in R version 3.6.0 (R Core Team, 2019).

## 3 RESULTS

### 3.1 Impact of urban cover, breeding date and rainfall on immune function and antioxidant capacity

**FIGURE 3:**
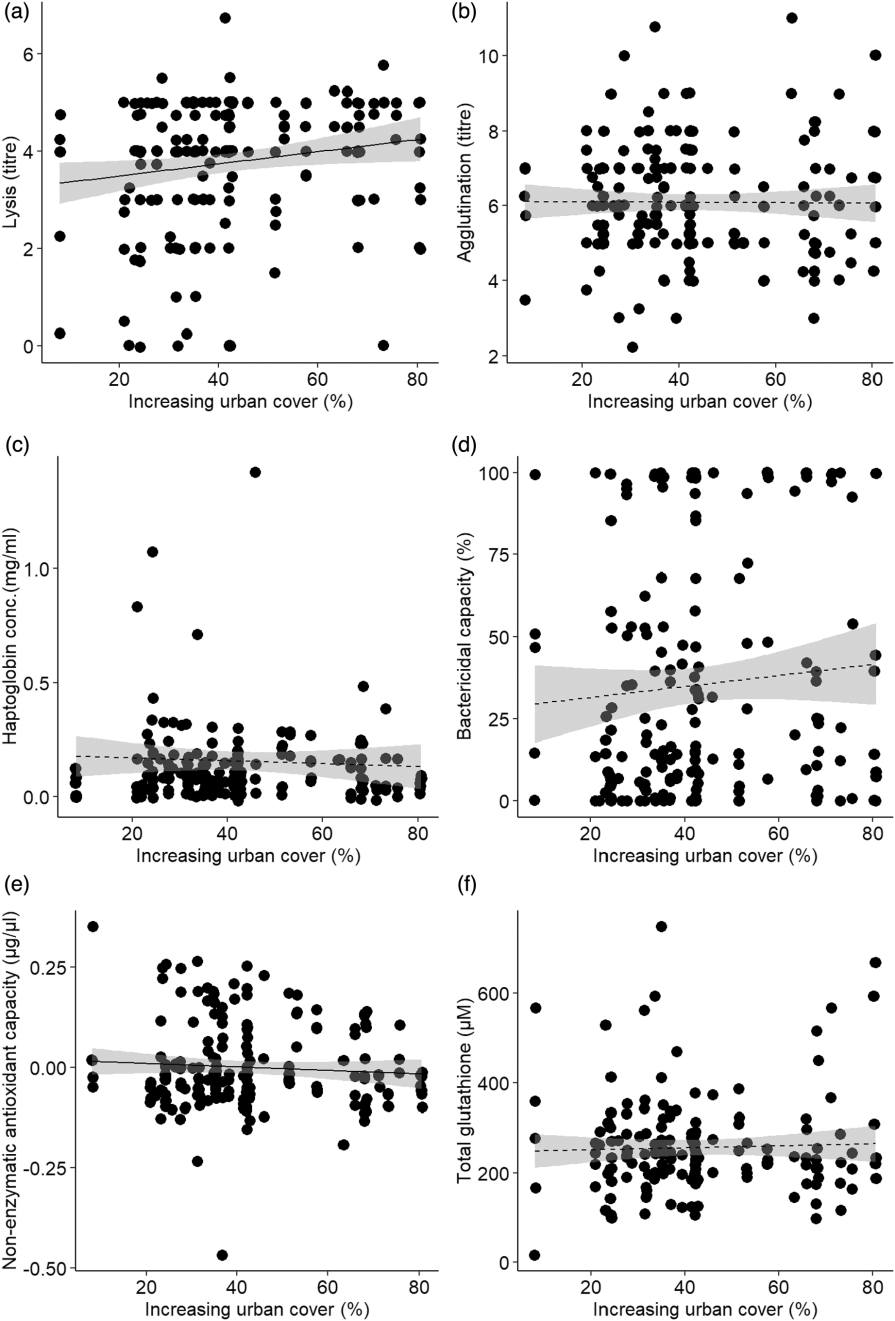
Relationship between urbanisation and indices of constitutive innate immune function as well as antioxidant capacity of nestling Black Sparrowhawks in Cape Town, South Africa. Correlation between urban cover and (a) lysis, (b) agglutination, (c) haptoglobin concentration, (d) bactericidal capacity (against *E. coli*), (e) non‐enzymatic antioxidant capacity corrected for uric acid and (f) total glutathione of nestlings. Solid/broken trend lines (with 95% confidence intervals) indicate significant or non‐significant relationships, respectively, from the model summaries reported in Table 2.

Lysis increased with increasing urban cover (Figure 3a, Table 2), but none of the other innate immune indices varied with urban cover (Figure 3b–d, Table 2). tAOX decreased with increasing urban cover (Figure 3e, Table 2), but tGSH did not vary with urban cover (Figure 3f, Table 2).

**TABLE 2:** A summary of linear mixed models showing variation in indices of constitutive immune function and antioxidant capacity in nestling Black Sparrowhawks. Birds were sampled on the Cape Peninsula, South Africa from 2015 to 2019. The relationship between urban cover, breeding date and rainfall, and immune function and antioxidant capacity were tested using separate linear mixed models. Additional explanatory variables include body mass index—Estimated as body mass‐tarsus length residual corrected for sex, sex and nestling age at sampling (centred days). Nestling age and rainfall (but their interaction with other variables) were retained in all models whether they were significant explanatory variable or not. All predictor variables in the full models are reported in the summary. Test statistics and associated *p*‐values for variables are for the best model or estimate at the time of elimination of a non‐significant variable or interaction from the model. Test statistics of main explanatory variables showing significant interactions with other variables are highlighted in grey because they cannot be correctly interpreted without their interaction terms. The model was simplified by stepwise backward elimination of non‐significant explanatory variables. Note that all quantitative variables were scaled. Non‐enzymatic antioxidant capacity (tAOX), total glutathione (tGSH)

Variable	*df*	Haemolysis			Haemagglutination			Haptoglobin			Bactericidal capacity			Non‐enzymatic AOC			Antioxidant activity of GSH		
		*F*	*p*		*F*	*p*		*F*	*p*		*F*	*p*		*F*	*p*		*F*	*p*	
Urban cover	1	**5.27**	**0.02**	*	0.00	0.99		0.64	0.43		1.52	0.22		**5.46**	**0.02**	*	0.80	0.37	
Rainfall	1	0.26	0.61		0.76	0.39		2.40	0.13		0.05	0.82		0.34	0.56		0.26	0.61	
Body mass index	1	0.12	0.73		1.30	0.26		0.10	0.75		1.28	0.26		0.01	0.93		0.22	0.64	
Sex	1	1.24	0.27		0.21	0.65		3.58	0.06		**11.00**	**<0.01**	**	0.06	0.81		**12.01**	**<0.01**	***
Age	1	**8.11**	**0.01**	**	0.00	0.98		0.07	0.79		**8.35**	**<0.01**	**	0.48	0.49		0.89	0.35	
Urban cover*Age	1	**6.20**	**0.01**	*	2.10	0.15		0.65	0.42		0.79	0.38		0.00	0.96		0.97	0.33	
Breeding date	1	**4.77**	**0.03**	*	0.03	0.86		1.69	0.20		2.05	0.16		0.41	0.53		0.99	0.32	
Breeding date^2	1	3.74	0.06								1.92	0.17							
Rainfall	1	1.51	0.22		0.45	0.51		0.18	0.68		1.34	0.25		0.13	0.72		0.10	0.76	
Body mass index	1	0.50	0.48		1.44	0.23		0.02	0.89		1.18	0.28		0.03	0.87		0.14	0.71	
Sex	1	0.39	0.53		0.08	0.78		4.17	0.04	*	**9.95**	**<0.01**	**	0.06	0.81		**10.54**	**<0.01**	***
**Age**	1	1.68	0.20		**6.01**	**0.02**	**	0.02	0.89		**8.75**	**<0.01**	**	0.54	0.46		1.30	0.26	
Breeding date*Age	1	1.30	0.26		**6.37**	**0.01**	**	2.09	0.15		0.82	0.37		0.03	0.86		0.41	0.53	

Agglutination increased with timing of breeding only after accounting for the association between breeding date and nestling age (Table 2), but none of the other innate immune indices, tAOX nor tGSH varied with timing of breeding (Figure S2A–F, Table 2).

Rainfall levels within 30 days before sampling were not associated with variation in innate immune indices, tAOX nor tGSH (Figure S3); however, lysis (Figure S2A) and bactericidal capacity (Figure S2D) varied with the timing of breeding in a pattern similar to the quadratic pattern of variation in rainfall across the breeding season (Figure S1).

### 3.2 Other factors: The impact of nestling age, sex and body mass index on immune function and antioxidant capacity

Lysis and bactericidal capacity were higher for older nestlings (Figure S4, Table 2 and Table 3). In addition, the relationship between lysis and urban cover, and agglutination, and the timing of breeding were only significant for older nestlings (Table 2). Male nestlings had lower haptoglobin concentration and lower bactericidal capacity compared to females, but lysis and agglutination did not differ between sexes (Table 2). Male nestlings had lower tGSH compared to females, but tAOX did not differ between sexes (Table 2). None of the immune indices, tAOX nor tGSH varied with body mass index (Table 2), but absolute uric acid concentration (used to correct FRAP) increased with body mass index (Figure S5).

**TABLE 3:** A summary of linear models showing variation in the immune response to a mimicked bacterial infection (lipopolysaccharide challenge), measured as the change in haptoglobin concentration over c.17 h, in nestling Black Sparrowhawks. Nestlings were challenged and sampled in either of 2018 or 2019 on the Cape Peninsula of South Africa. We built separate models to test the relationship between immune response and urban cover, breeding date and rainfall due to collinearity between these explanatory variables. Other explanatory variables include body mass index—calculated as body mass‐tarsus length residual corrected for sex, sex, nestling age at sampling (centred days) and haptoglobin concentration before immune challenge (pre‐challenge Hp). The model was simplified by stepwise backward elimination of non‐significant explanatory variables. All predictor variables in the full models are reported in the summary. Test statistics and associated *p*‐values for variables are for the best model or estimate at the time of elimination from the model. Test statistics of main explanatory variables showing significant interactions with other variables are highlighted in grey because they cannot be interpreted without the interaction terms. Note that all quantitative variables were scaled

	Factor	*df*	*F*	*p*	
Urban cover model	Urban cover	1	0.12	0.73	
	Rainfall	1	0.21	0.65	
	Body mass index	1	0.14	0.71	
	Sex	1	0.21	0.65	
	Age	1	1.89	0.18	
	Pre challenge Hp	1	7.75	<0.01	**
	Urban cover*Age	1	8.88	<0.01	**
Breeding date model	Breeding date	1	0.61	0.44	
	Rainfall	1	0.00	0.98	
	Body mass index	1	0.86	0.36	
	Sex	1	0.14	0.71	
	Age	1	1.96	0.17	
	Pre challenge Hp	1	8.69	<0.01	**
	Breeding date*Age	1	2.24	0.14	

### 3.3 Impact of urban cover, breeding date and rainfall on the immune response to a mimicked bacterial infection

**FIGURE 4:**
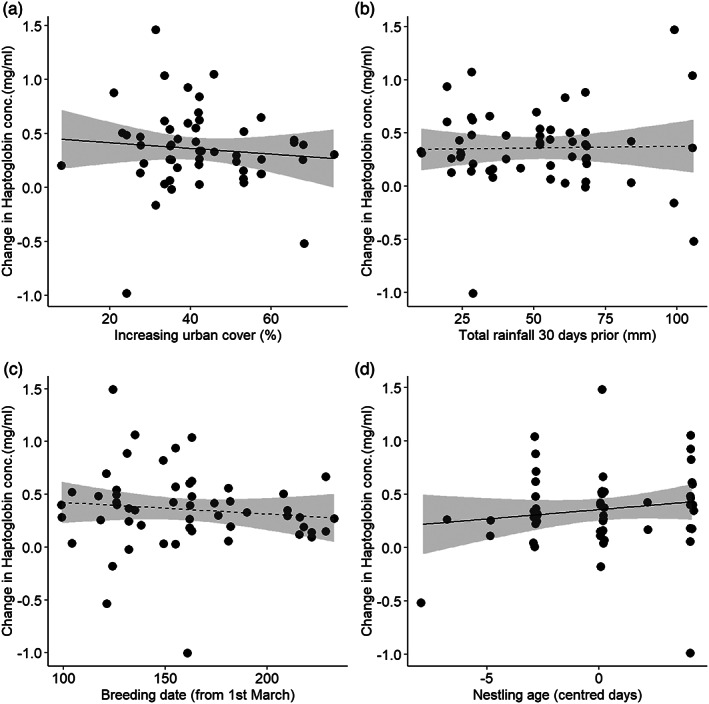
Correlation between change in haptoglobin concentration after lipopolysaccharide challenge and (a) urban cover across breeding territories, (b) average daily rainfall over 30 days prior to sampling (c) breeding date and (d) nestling age of Black Sparrowhawk at sampling. Solid/dashed trend lines (with 95% confidence intervals) indicate significant or non‐significant relationships, respectively, from the model summaries reported in Table 2.

Change in haptoglobin concentration after the mimicked bacterial infection decreased with increasing urban cover (Figure 4a, Table 3), but was not associated with the timing of breeding nor rainfall (Figure 4b,c, Table 3). Change in haptoglobin concentration after LPS injection increased with nestling age (Figure 4d) and with haptoglobin concentration prior to the LPS injection but did not differ between male and female nestlings (Table S3). Furthermore, the relationship between change in haptoglobin concentration after LPS injection and urban cover did not depend on sex (*F*_1_, _47_ = 0.002, *p* = 0.76). Similarly, haptoglobin concentration pre‐LPS‐injection did not differ between sexes (*F*_1_, _52_ = 2.41, *p* = 0.13).

### 3.4 Hypothetical indirect association between urban cover and immune function

**FIGURE 5:**
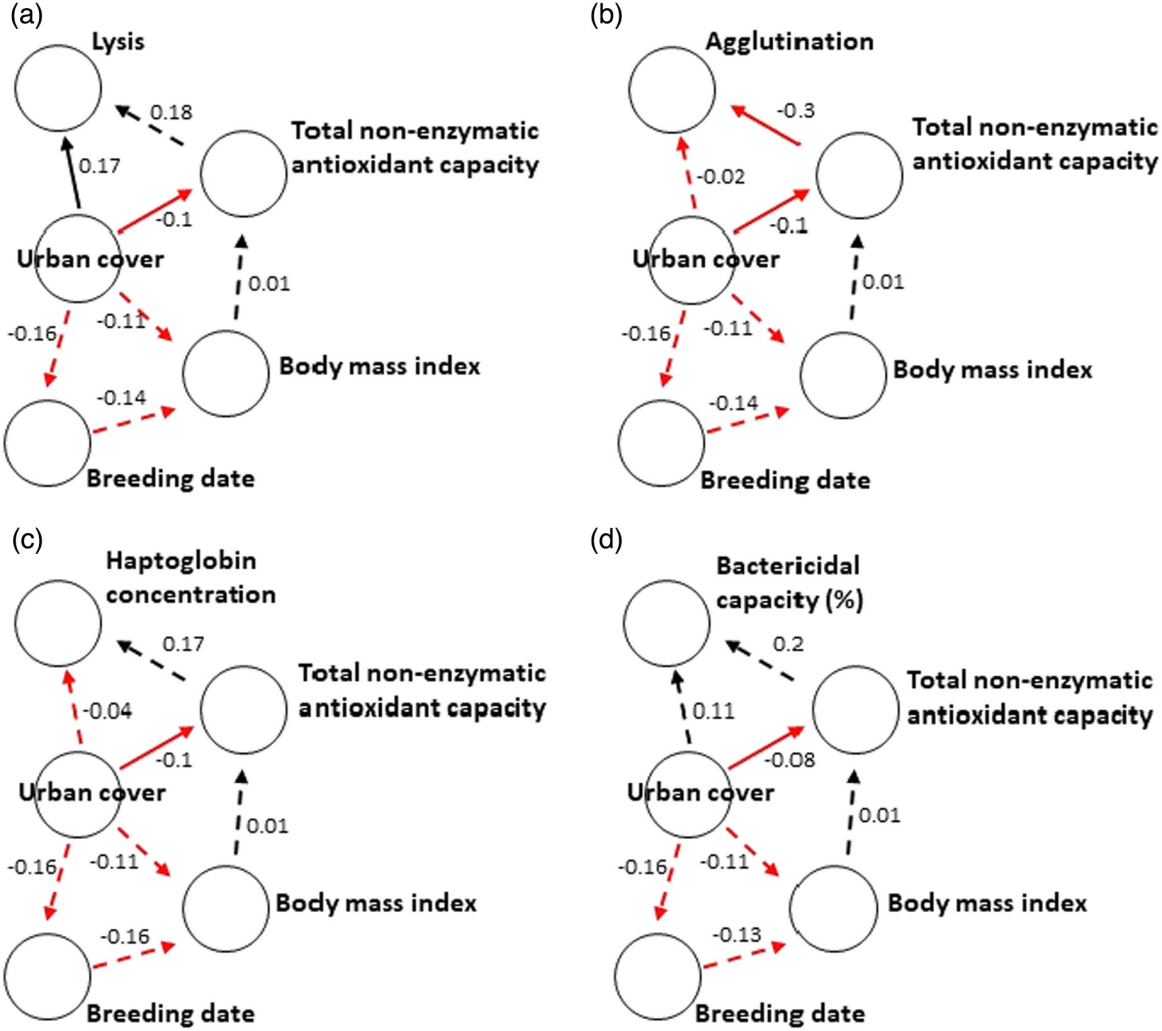
Rudimentary path diagrams showing hypothetical direct and indirect connection between urban cover and (a) lysis, (b) agglutination, (c) haptoglobin concentration and (d) bactericidal capacity (against *E. coli*) via total non‐enzymatic antioxidant capacity of nestling Black Sparrowhawk sampled along a gradient of varying urban cover, assuming antioxidant capacity affects immune function. Arrows indicate direction of potential effect. Non‐significant and marginal relationships are shown as dashed lines. Red arrows and negative numbers indicate negative effects, while black arrows and positive numbers indicate positive effects. Standardised estimates from structural equation models showing the strength of the relationship between variables are indicated with each arrow. Overall, there was an indirect connection between urban cover and agglutination via antioxidant capacity, but urban cover was directly associated with antioxidant capacity and lysis but not haptoglobin concentration, agglutination and bactericidal capacity. There was no support for an indirect association between urban cover and immune indices via timing of breeding, body mass index and total non‐enzymatic antioxidant capacity.

**FIGURE 6:**
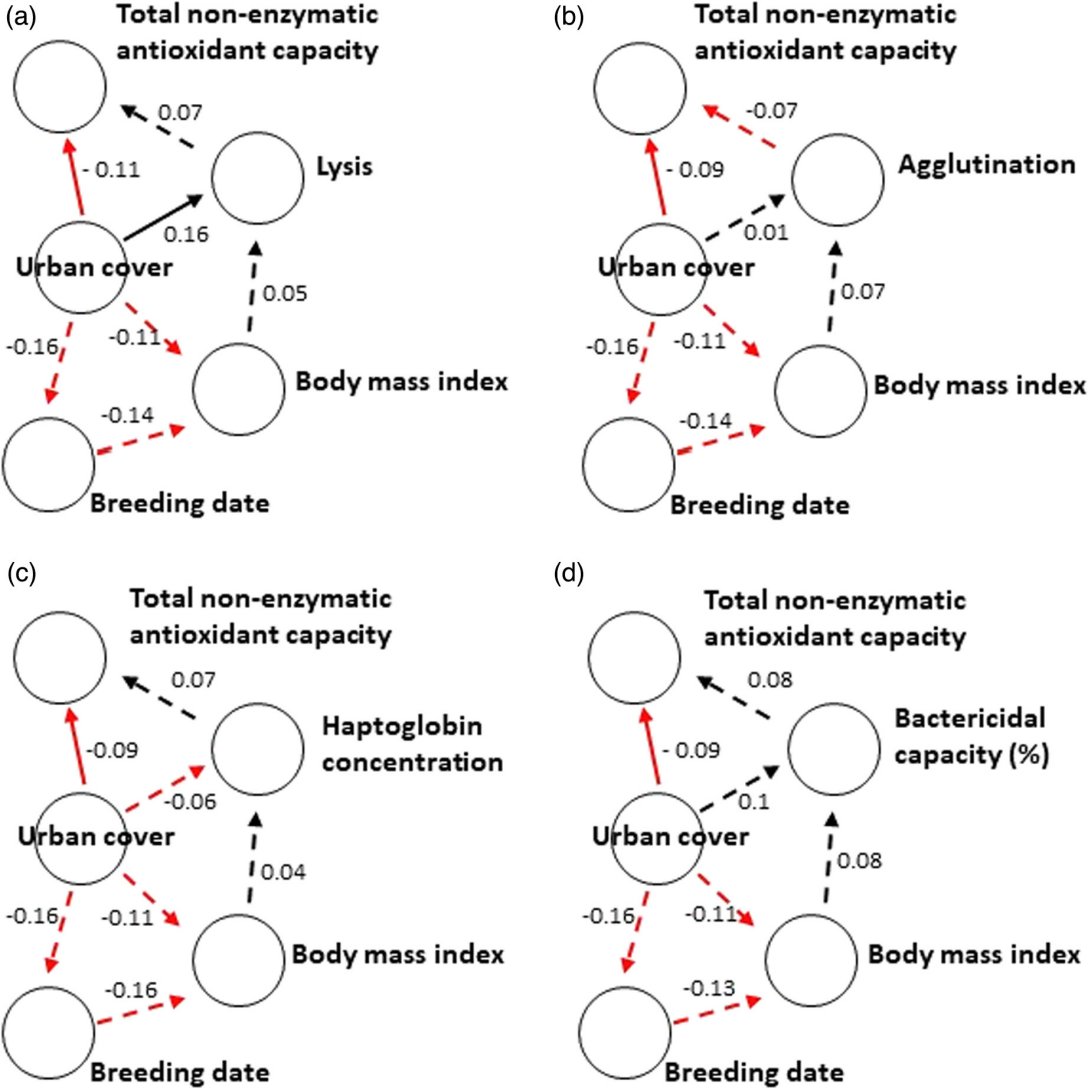
Rudimentary path diagrams showing hypothetical direct and indirect connections between urban cover and antioxidant capacity via (a) lysis, (b) agglutination, (c) haptoglobin concentration and (d) bactericidal capacity (against *E. coli*) of nestling Black Sparrowhawk, assuming immune function affects antioxidant capacity. Arrows indicate direction of potential effect. Non‐significant relationships are shown as dashed lines. Red arrows and negative numbers indicate negative effects, while black arrows and positive numbers indicate positive effects. Standardised estimates from structural equation models showing the strength of the relationship between variables are indicated next to each arrow. Overall, there was no indirect connection between urban cover and antioxidant capacity via lysis, agglutination, haptoglobin concentration and bactericidal capacity. Urban cover was, however, directly associated with antioxidant capacity and lysis. There was no support for an indirect connection between urban cover and total non‐enzymatic antioxidant capacity via the timing of breeding, body mass index and immune indices.

The path analyses showed that only lysis and tAOX were directly and independently associated with urban cover (Figure 5a), whereas agglutination was more likely to be associated with urban cover via tAOX (Figure 5b). The associations between tAOX and lysis, agglutination, haptoglobin concentration and bactericidal capacity recorded higher effect sizes irrespective of their significance, suggesting that these immune indices were more likely to be influenced by urban cover via tAOX (Figure 5a–d). Urban cover was unlikely to be associated with innate immune indices via body mass index (Figure 5a–d). However, the association between urban cover and the timing of breeding (pairs in more urbanised territories bred earlier) and timing of breeding and body mass index (nestlings raised later in the breeding season have a lower body mass index) were relatively strong. Body mass index was, however, not associated with tAOX (Figure 5a–d). The alternative pathway (Figure 5), of immune function predicting tAOX, was not better supported than the hypothesis that urbanisation influences immune function via tAOX (Figure 6, Table 4: 2 < delta AIC < 4 [Burnham & Anderson, 2004]). While tAOX predicted lysis, agglutination, haptoglobin concentration and bactericidal capacity with high effect sizes when urban cover was included in our models (Figure 5a–d), lysis, agglutination, haptoglobin concentration and bactericidal capacity did not predict tAOX with similar effect sizes when urban was cover included in our models (Figure 6a–d).

**TABLE 4:** Comparison of alternative hypothetical pathways for the association between urbanisation, immune function and antioxidant capacity. A—Urban cover affects indices of innate constitutive immune function via impact on antioxidant capacity (Figure 4), B—Urban cover affects antioxidant capacity via impact on immune function (Figure 5). In both cases, we further tested the association between urban cover, timing of breeding, body mass index, immune function and antioxidant capacity. Overall, there is no strong evidence against model A since 2 < deltaAIC < 4

Immune index		*df*	AIC	deltaAIC
Haptoglobin concentration	A	19	53.79	0.46
	B		53.33	
Lysis	A		53.02	2.16
	B		50.86	
Agglutination	A		50.36	2.35
	B		48.01	
Bactericidal capacity	A		51.39	2.64
	B		48.74	

## 4 DISCUSSION

This study investigated the impact of urbanisation on aspects of constitutive innate immune function, innate immune responses and antioxidant capacity of nestling Black Sparrowhawks on the Cape Peninsula, South Africa and found a direct association between urbanisation and aspects of both immune function in unchallenged individuals and its response to a mimicked bacterial infection, and antioxidant capacity. We also found an indirect association between urbanisation and innate immune function via total non‐enzymatic antioxidant capacity.

Lysis, a measure of compliment activity, was the only innate immune index that varied with urbanisation, and its observed increase with urban cover suggests that nestlings in more urbanised territories may have expressed a stronger capacity to fight infection. Alternatively, it may be related to current infection rates which may be higher in more urban territories because lysis and bactericidal capacity also increased with the timing of breeding in a manner similar to variation in rainfall—a proxy of infection risk in wet–dry seasonal environments (Altizer et al., 2006; Nwaogu et al., 2019; Pascual et al., 2002; Pascual & Dobson, 2005; Tieleman et al., 2019). Higher levels of innate immune function should benefit nestlings if they are more likely to be infected in more urban territories (Bradley & Altizer, 2007), but it is not clear whether the observed high lysis is in response to current infection or an adaptation to the general risk of infection in this environment. A previous study in our population found that the risk of infection by *Leucocytozoon toddi* declines with increasing urban cover (Suri et al., 2017), but it is still valid to assume that other types of infections can be higher in more urban territories, because environmental productivity may influence other potentially virulent pathogens in different ways. For example, while haemoparasite infection tends to decrease with increasing urbanisation (Bailly et al., 2016; Evans et al., 2009; Fokidis et al., 2008; Suri et al., 2017), the prevalence of coccidia and poxvirus (Giraudeau et al., 2014) and trichomoniasis (Boal et al., 1998; Boal & Mannan, 2000; Rosenfield et al., 2002) increased with urbanisation in other species. Black Sparrowhawks in our study area are sometimes infected by *Knemidokoptes* mites (van Velden et al., 2017) which can trigger inflammatory response, but there is no indication that this infection is associated with urbanisation. The patterns of variation in immune indices do not suggest whether urbanisation improves the condition of Black Sparrowhawks or not. Regardless, whether higher levels of immune indices in more urban territories serve to protect nestlings against current infection or not, upregulation of immune function in more urban territories may entail physiological costs (Hasselquist & Nilsson, 2012) with life‐history implications that depend on whether upregulation of immune function improves survival (Eraud et al., 2009; Møller & Saino, 2004; Wilcoxen et al., 2010).

In contrast to our prediction, nestlings from the more urbanised sites had lower antioxidant capacity. The opposite pattern has been shown in other species (Herrera‐Dueñas et al., 2017; Hõrak et al., 2010; Salmón et al., 2018), suggesting that urbanisation may impact antioxidant capacity in both directions, possibly due to differences among species, type of urban environment or depending on the level of urbanisation and exposure to urban stressors (Isaksson, 2020). Note that we only found a negative relationship between urban cover and antioxidant capacity with tAOX but not with tGSH. tAOX is affected by the systemic physiological and nutritional state, while tGSH is an endogenously synthesised antioxidant (Isaksson et al., 2011). Glutathione, however, is part of a redox cycle, where its used form (oxidised, GSSG) is recycled back to its active form. Due to transport delays and the sensitivity of the assay, we were unable to reliably measure GSSG, and were thus not able to assess their redox state. Nonetheless, the absolute levels of tGSH can also be influenced by several environmental factors related to urbanisation such as exposure to heavy metals (Jozefczak et al., 2012; Rubino, 2015), thermal‐stress (Ohtsuka et al., 1994) and starvation (Cho et al., 1981). Possibly, due to its importance and because glutathione is genetically regulated, urban nestlings may keep their tGSH levels high by continually generating new GSH. Instead, the more multifaceted tAOX shows environmental variability that may reflect the challenges of urban life and incapability to upregulate the defences, which could be detrimental in the long term. Alternatively, one can argue that urban nestlings do not experience a pro‐oxidative challenge; hence, they show lower tAOX compared to less urbanised broods. To distinguish between these two opposing explanations, a marker of oxidative damage is required, which unfortunately could not be measured here.

The capacity of Black Sparrowhawk nestlings to respond to a mimicked bacterial infection decreased with increasing urban cover, although this relationship was only visible in older nestlings. Great Tit *Parus major* nestlings have also been shown to have a weaker response to an immune challenge in more urbanised habitats (Bailly et al., 2016), and an altered immune response when exposed to artificial light at night (Ziegler et al., 2021), suggesting that variation in immune function along an urban gradient may have a functional significance. Interestingly, this decreasing immune response to a mimicked bacterial infection contrasts the pattern observed for variation in lysis which increased with urban cover. This contrast reinforces the notion that immune indices from unchallenged individuals and a response to a mimicked infection are not necessarily correlated and can have different patterns of variation. This difference is likely due to their different costs and functions (Hegemann et al., 2013; Vermeulen et al., 2016; Vinterstare et al., 2019). For example, variation in haptoglobin concentration, our index of immune response to a mimicked bacterial infection has numerous functions, including resistance and tolerance to infection. Haptoglobin concentration has been shown to correlate negatively with lysis, body mass and onset of moult in birds under diet restriction (Nwaogu, Galema, et al., 2020). It also correlates negatively with Immunoglobulin Y (IgY)—another index of immune function associated with disease resistance, in wild birds infected with avian malaria (Arriero et al., 2018). Therefore, decreasing haptoglobin concentration with increasing urban cover cannot be solely interpreted as evidence of an impaired immune response to infection. It may also imply that Black Sparrowhawk nestlings in more urban territories adopt an alternative response to infection depending on their condition. Consistently, the opposite pattern between lysis in unchallenged birds and haptoglobin response to a mimicked bacterial infection in this study suggests different strategies by urban versus less urban birds.

The different patterns of variation highlight the importance of measuring several indices of immune function when seeking to draw general conclusions about variation in immune function. Other studies that investigated the impact of urbanisation or other stressors that affect nestling immune function either measured only innate immune indices without administering immune challenges (Merrill et al., 2019; Raap et al., 2017; Roncalli et al., 2018, 2020) or only quantified immune responses to a mimicked infection without measuring constitutive immune function (Bailly et al., 2016), but not both (for an exception see Ziegler et al., 2021). By measuring both aspects of innate immune function in this study, we can more conclusively report that variation in urban cover impacts aspects of both innate immune function in unchallenged individuals and immune responses to a mimicked bacterial infection in Black Sparrowhawk nestlings, albeit in opposite directions. Our results suggest hidden associations between urbanisation and innate immune function. Path analyses revealed that urbanisation was positively (agglutination) and negatively (lysis and bactericidal capacity) associated with innate immune function indirectly via its negative impact on tAOX. The association between urbanisation and immune function is therefore not limited to visible correlations between urban cover and immune indices. Relationships between antioxidant capacity and immune function have previously been suggested (Catoni et al., 2008; Cram et al., 2015; Eikenaar et al., 2018, 2020). For example, bactericidal capacity and tAOX were negatively correlated in a migratory songbird *Turdus merula* (Eikenaar et al., 2018), possibly indicating a trade‐off between maintenance of antioxidant capacity and immune function during migration. The relationships between antioxidant capacity and our immune indices are unlikely to be limited to trade‐offs: while agglutination decreased with increasing tAOX, lysis and bactericidal capacity increased, suggesting that antioxidant capacity (and/or oxidative stress) may be indirectly associated with the upregulation of immune function (Hasselquist & Nilsson, 2012). However, the limited support for the alternative hypothetical pathway, that is, that urbanisation influences tAOX via immune function, suggests that a direct rather than an indirect association between urbanisation and tAOX is more likely. It is, therefore, likely that urbanisation impairs aspects of innate immune function by limiting the capacity of nestlings to prevent oxidative stress. One would expect high‐‘quality’ nestlings to show stronger antioxidant capacity, lysis and bactericidal capacity, but curiously, none of these indices were correlated with body mass index. In a related study (Nebel et al., 2021), nestlings with lower body mass index showed higher survival rates—an index of energy reserves and physical well‐being. This finding raises questions about the suitability of avian body morphometrics (Green, 2001; Peig & Green, 2010) as proxies of individual condition. It, however, stresses the importance of incorporating other life‐history traits such as immunocompetence and antioxidant capacity when seeking to understand the impact of environment on individual condition. Nonetheless, body mass index may associate with other physiological traits or nestlings may adopt alternative strategies to balance oxidative capacity depending on their condition. For example, uric acid, a product of protein breakdown has been suggested to be retained in the body for a longer time as a cheaper way to boost antioxidant defences (Eikenaar et al., 2016). Here, uric acid correlates strongly with body mass index (Figure S5), suggesting that those nestlings that have a high body mass index have digested more protein and subsequently increased uric acid‐based antioxidant protection or that lower body mass individuals require lower antioxidant levels.

Innate immune indices and immune response to a mimicked bacterial infection were age dependent, consistent with suggestions that some components of the innate immune system develop later in the nestling phase (Aastrup & Hegemann, 2021; Killpack et al., 2013; Killpack & Karasov, 2012; Palacios et al., 2009). Apparently, younger nestlings were unable to respond to the immune challenge in the same way as older ones, but since nestling age did not correlate with urban cover in our data (Table S3), variation in nestling age should not affect the overall outcome of the study, rather it reflects the higher vulnerability of young animals to infection.

To conclude, our findings present a new viewpoint from which the impact of urbanisation on wildlife can be considered, that is, through the impact of urbanisation on phenology and the associations between breeding phenology, physiology and seasonal environmental change. We provide evidence that early‐life development in an urban environment has multiple physiological impacts on nestlings. The direct association between urbanisation and antioxidant capacity and their impact on immune function is likely a crucial factor mediating the impact of urbanisation on urban‐dwelling animals. Unprecedented urban expansion can have severe fitness consequences for urban animals because early‐life immune function and antioxidant capacity are linked to long‐term survival (Saino et al., 2011).

## AUTHOR CONTRIBUTIONS

Chima Josiah—fieldwork, data analyses and first draft; Arjun Amar—study design, fieldwork and editing; Carina Nebel—fieldwork, laboratory analyses and editing; Caroline Isaksson—study design, laboratory analyses and editing; Arne Hegemann—study design, laboratory analyses and editing; Petra Sumasgutner—study design, fieldwork, laboratory analyses and editing.

## CONFLICT OF INTEREST

Authors declare no conflict of interest.

## Supporting information


Data S1

## Data Availability

Data are available from the Dryad Digital Repository https://doi.org/10.5061/dryad.31zcrjdjp (Nwaogu et al., 2022).
