# Supplementary material for: Innate immune function and antioxidant capacity of nestlings of an African raptor covary with the level of urbanisation around breeding territories
Source: J Anim Ecol. 2022 Dec 4;92(1):124–41. doi: 10.1111/1365-2656.13837 (PMC10107107; doi:10.1111/1365-2656.13837)
Supplement: Supplementary file 1 — Data S1 [file JANE-92-124-s001.docx]

**Online supporting information**

**Innate immune function and antioxidant capacity of nestlings of an African raptor co-vary with the level of urbanisation around breeding territories**

Chima Josiah Nwaogu^1^*, Arjun Amar^1^, Carina Nebel^12^, Caroline Isaksson^3^, Arne Hegemann^3♱^, Petra Sumasgutner^1,4,5♱^

^1^ FitzPatrick Institute of African Ornithology, DST-NRF Centre of Excellence, University of Cape Town, Cape Town, South Africa

^2^ Department of Biology, University of Turku, Turku, Finland

^3^ Department of Biology, Lund University, Lund, Sweden

^4^ Konrad Lorenz Research Centre, Core Facility for Behaviour and Cognition, University of Vienna, Vienna, Austria

^5^ Department of Behavioural & Cognitive Biology, University of Vienna, Vienna, Austria

* Corresponding author: [chima.nwaogu@uct.ac.za](mailto:chima.nwaogu@uct.ac.za)

♱ Joint senior authors

**ORCID:** Nwaogu Chima Josiah [0000-0002-4623-2355](https://orcid.org/0000-0002-4623-2355); Arjun Amar 0000-0002-7405-1180; Carina Nebel: 0000-0002-0848-1676; Caroline Isaksson: 0000-0002-6889-1386; Arne Hegemann: 0000-0002-3309-9866; Petra Sumasgutner 0000-0001-7042-3461


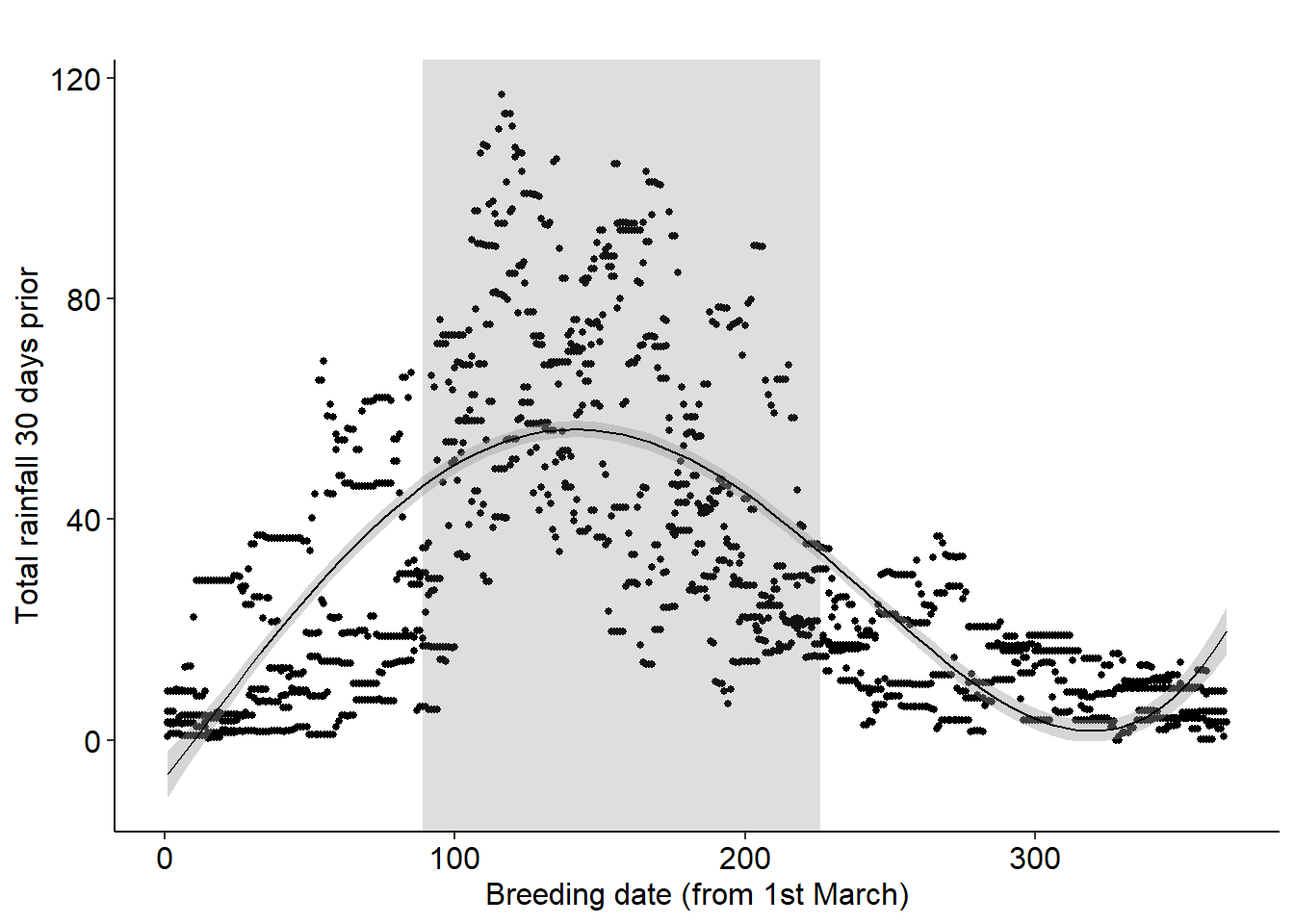


**Figure S1:** Running total of daily rainfall over a 30-day period showing seasonal rainfall pattern on the Cape Peninsula, South Africa. Rainfall data was obtained from the South African Weather Service station located at the Cape Town Airport (33.96, 18.60, altitude: 42 m). The weather station is located c.16 km from the central point of our study area. The periods when nestlings were sampled for this study are highlighted in grey. The long-term start of egg laying for this population is day 0 (c. 1^st^ March), incubation lasts on average 38 days and nestlings fledge c. 45 days after hatching (Steyn, 1982). Thus, the earliest nestlings in the season are sampled 80 - 90 days after March 1^st^. Trend line presented with 95% confidence intervals (grey band).


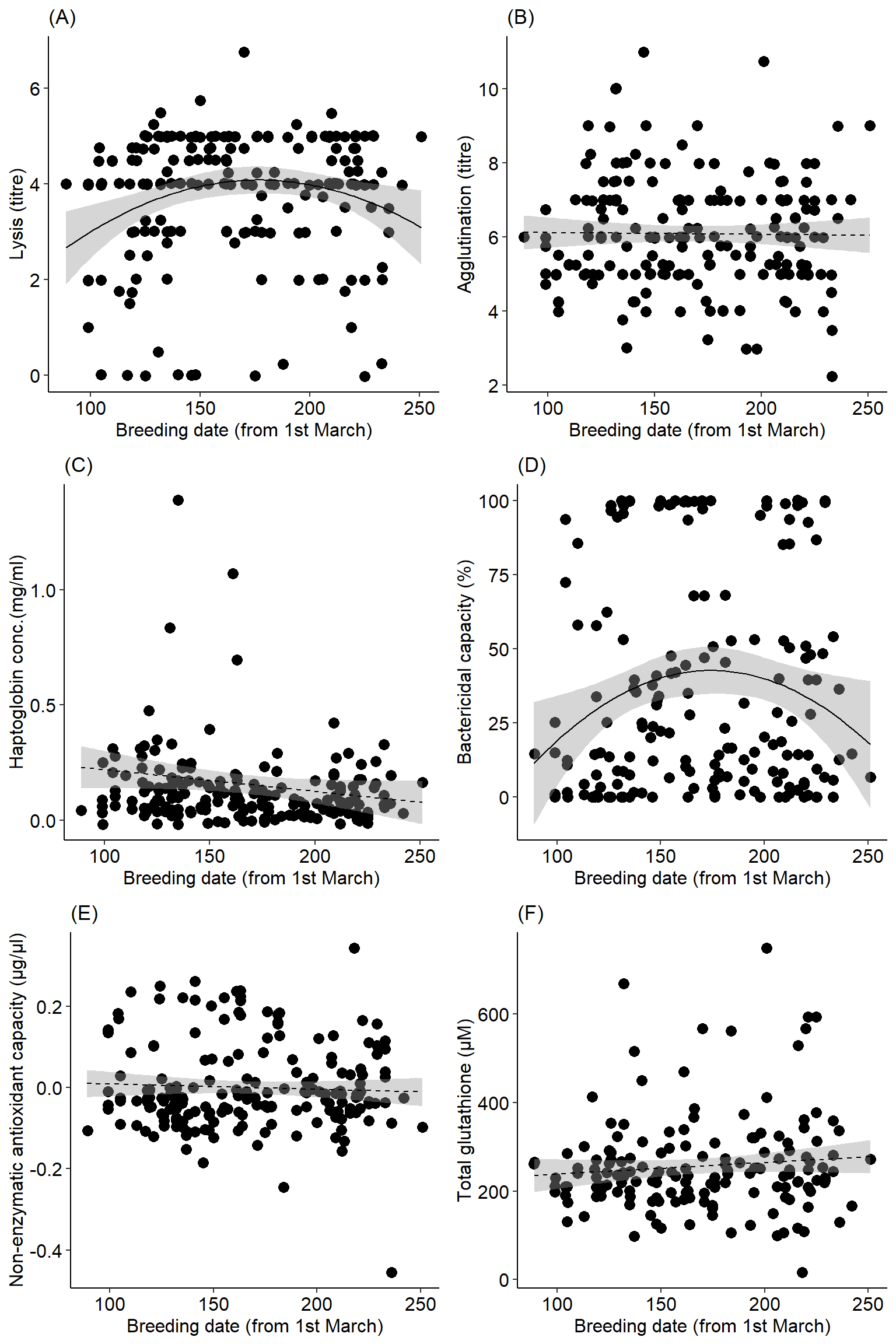


**Figure S2:** Relationship between timing of breeding date and innate immune function as well as antioxidant capacity of nestling Black Sparrowhawks in Cape Town, South Africa. Correlation between breeding date and (A) lysis, (B) agglutination, (C) haptoglobin concentration, (D) bactericidal capacity (against *E. coli*), (E) non-enzymatic antioxidant capacity, and (F) total glutathione of nestlings sampled across breeding territories with varying urban cover in Cape Town, South Africa. Dashed lines indicate statistically non-significant relationship in model summaries reported in table 1. Trend line presented with 95% confidence intervals (grey band).


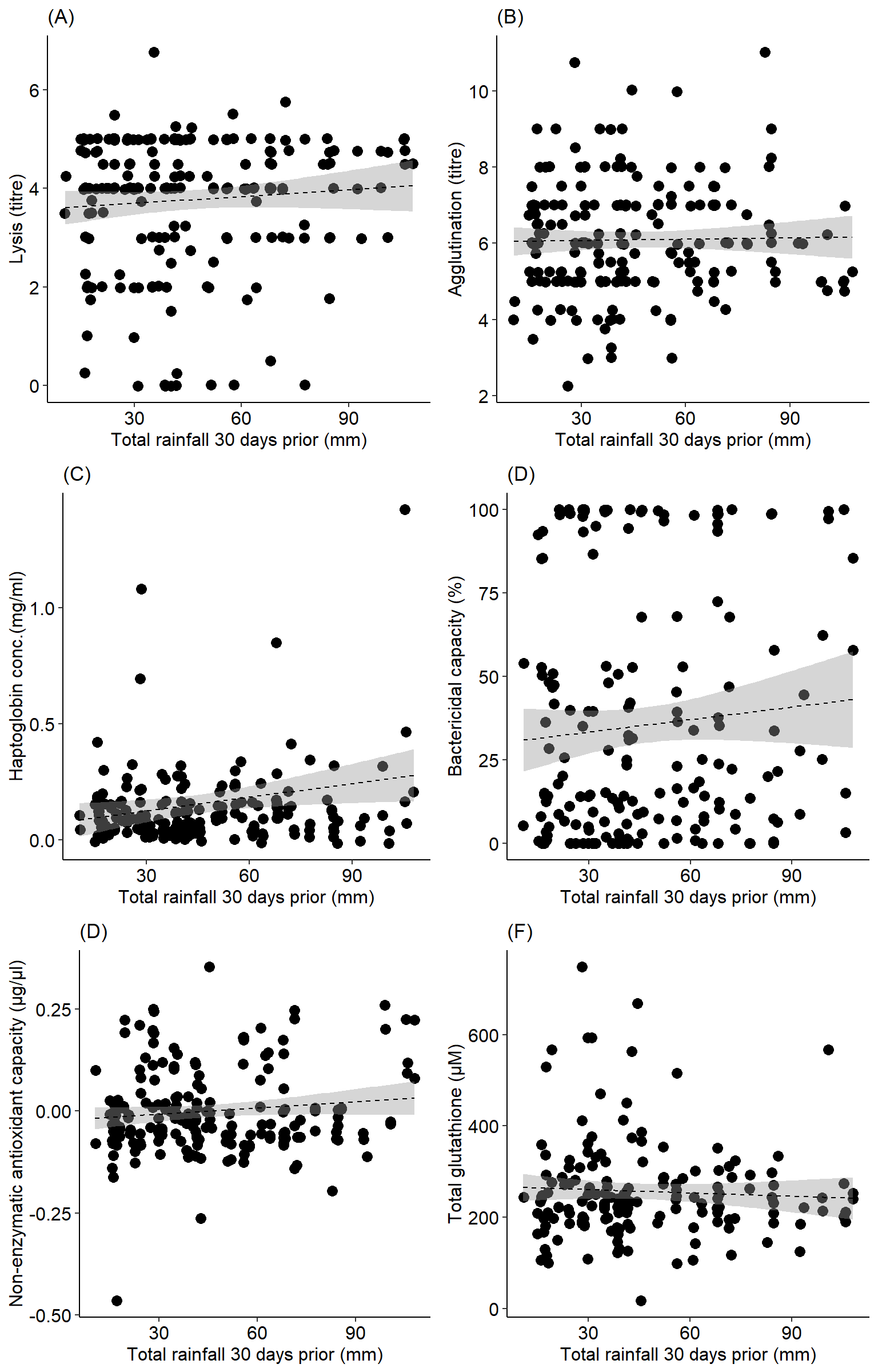


**Figure S3:** Relationship between rainfall and innate immune function as well as antioxidant capacity of nestling Black Sparrowhawks over the breeding season. Correlation between log total daily rainfall over 30 days and (A) lysis, (B) agglutination, (C) haptoglobin concentration, (D) bactericidal capacity (against *E. coli*), (E) non-enzymatic antioxidant capacity, and (F) total glutathione of nestlings sampled across breeding territories with varying urban cover on the Cape Peninsula, South Africa. Dashed lines indicate statistically non-significant relationship in model summaries reported in table 1. Trend line presented with 95% confidence intervals (grey band).


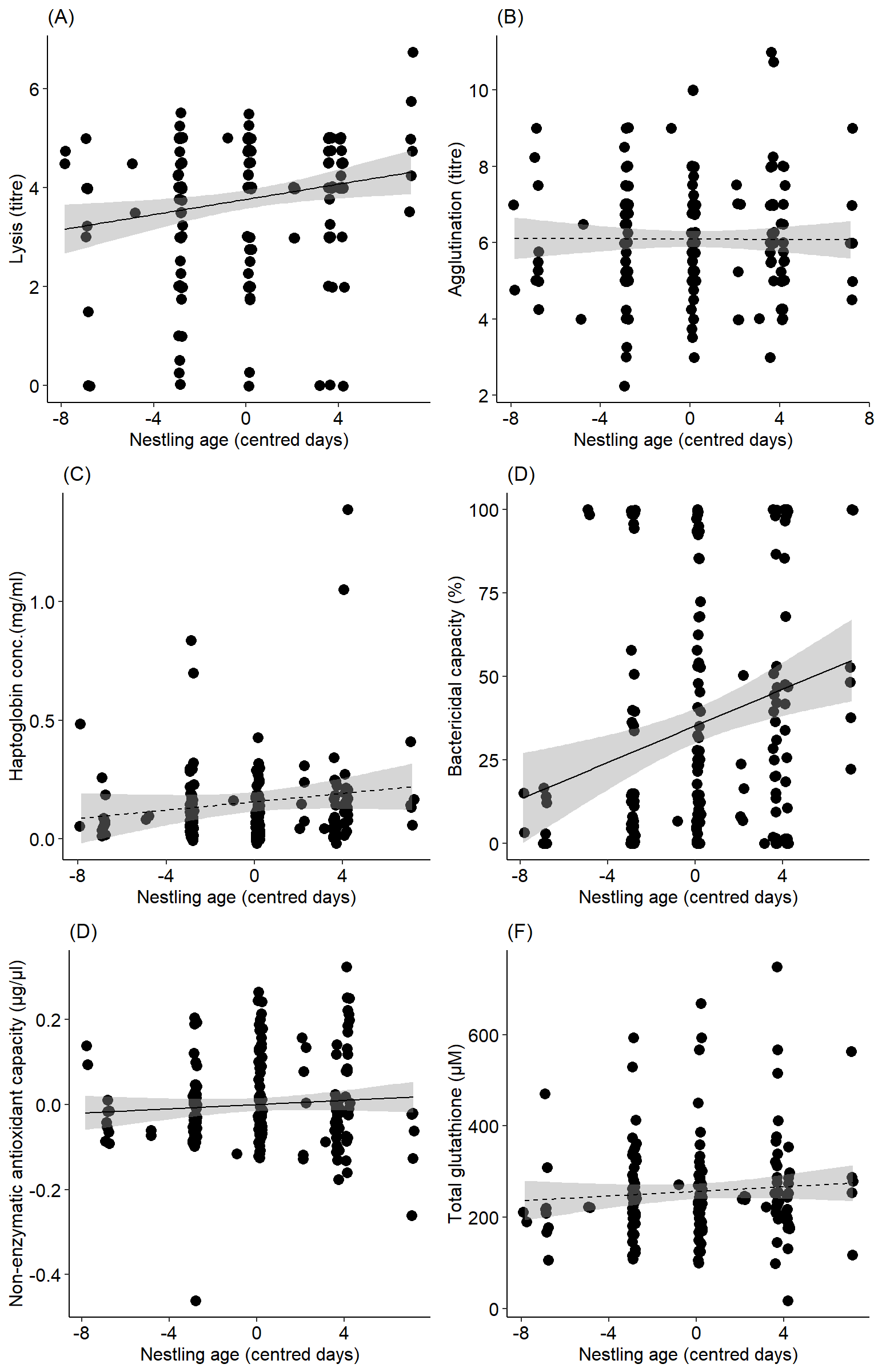


**Figure S4:** Relationship between nestling age and indices of innate immune function and antioxidant capacity in Black Sparrowhawks from the Cape Peninsula, South Africa. Correlation between nestling age and (A) lysis, (B) agglutination, (C) haptoglobin concentration, (D) bactericidal capacity (against *E. coli*), (E) non-enzymatic antioxidant capacity, and (F) total glutathione of nestlings sampled across breeding territories with varying urban cover. Dashed lines indicate statistically non-significant relationships in model summaries reported in table 1. Trend line presented with 95% confidence intervals (grey band).


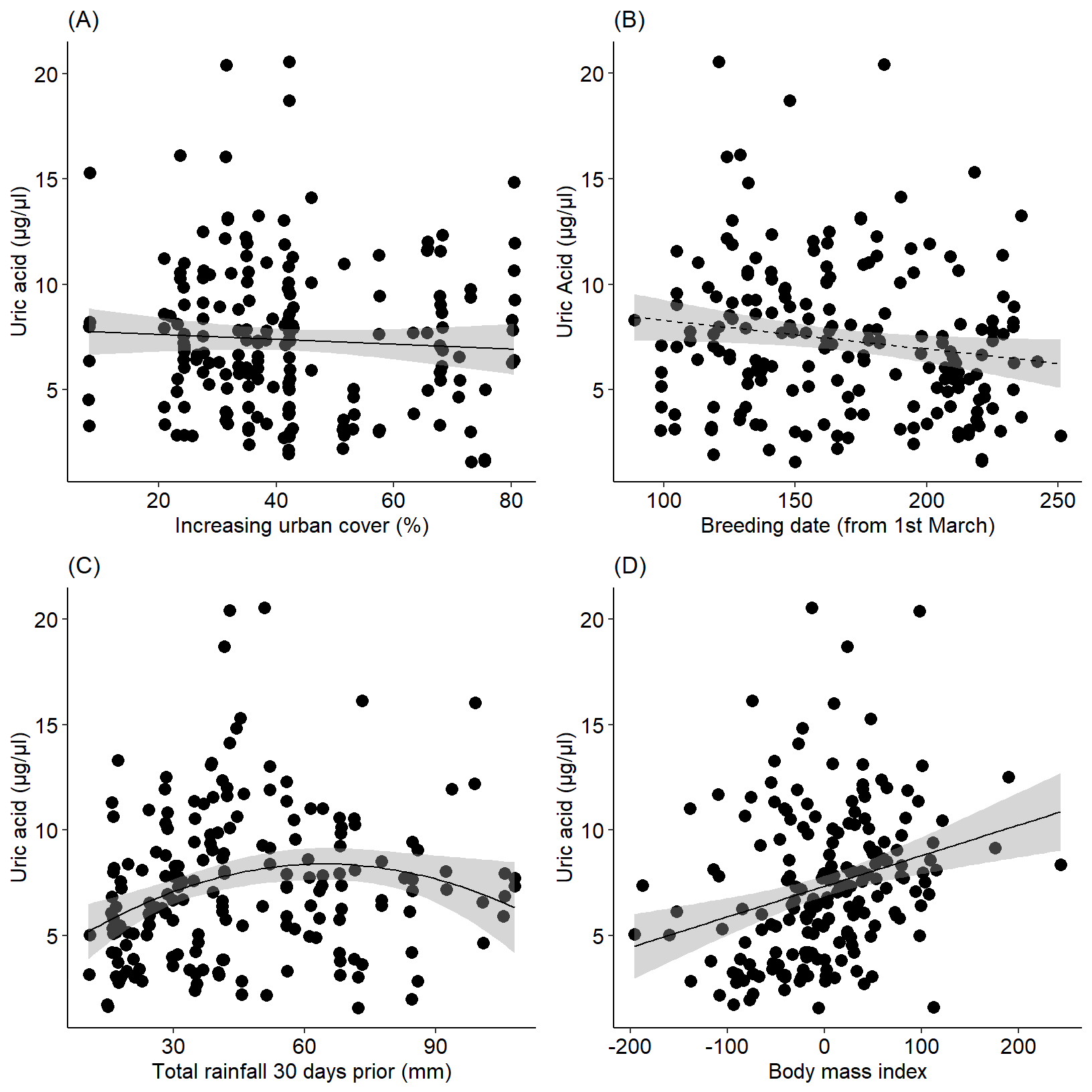


**Figure S5:** Plasma uric concentration decreases with urban cover and increases with rainfall and body mass index, but not with the timing of breeding in nestling Black Sparrowhawks on the Cape Peninsula, South Africa. Correlation between uric acid and (A) urban cover, (B) breeding date, (C) average rainfall, and (D) body mass index of nestling Black Sparrowhawk sampled in breeding territories with varying urban cover. Dashed lines indicate statistically non-significant relationships in model summaries reported in table 1. Trend line presented with 95% confidence intervals (grey band).

**Table S1**: Sampling locations of Black Sparrowhawks around the Cape Peninsula, South Africa with number of samples collected per year and territory. Territories are arranged in increasing urban cover (for definition see main text). Cells highlighted in grey in 2018 and 2019 are territories where one or two nestlings were immune challenged with lipopolysaccharide to measure an immune response. Table shows totals from nests where information on nestling age, body mass, tarsus length, and sex were available, and where the blood sample was sufficient to carry out the haemolysis and agglutination assay (200 samples). Analyses for the other immune assays and indices of antioxidant capacity had slightly smaller sample sizes due to plasma limitations: haptoglobin concentration (194 samples), bacteria killing capacity (183 samples), total non-enzymatic antioxidant capacity (192 samples), uric acid (192 samples), total glutathione (164 samples) and immune response to lipopolysaccharide injection (64 samples).

|  |  |  |  | **Sample size** | | | | | |
| --- | --- | --- | --- | --- | --- | --- | --- | --- | --- |
| **Territory** | **longitude** | **latitude** | **urban cover** | **2015** | **2016** | **2017** | **2018** | **2019** | **Total** |
| Orange Kloof | 18.40 | -34.00 | 8.10 | 1 | 2 | 2 | 1 | 0 | 6 |
| Athlone | 18.38 | -34.02 | 20.91 | 0 | 0 | 3 | 0 | 2 | 5 |
| Newlands 2-3 | 18.44 | -33.97 | 21.95 | 0 | 0 | 2 | 0 | 0 | 2 |
| Newlands 1-6 | 18.45 | -33.97 | 23.11 | 1 | 0 | 2 | 1 | 0 | 4 |
| Southern Cross Drive | 18.42 | -34.01 | 23.56 | 0 | 1 | 0 | 2 | 0 | 3 |
| Imhoff 2-2 | 18.35 | -34.14 | 24.18 | 0 | 2 | 2 | 0 | 1 | 5 |
| Meadowridge | 18.42 | -34.06 | 24.24 | 3 | 2 | 2 | 1 | 0 | 8 |
| Imhoff 1 | 18.34 | -34.14 | 25.72 | 1 | 0 | 0 | 0 | 0 | 1 |
| De Hel 1 | 18.42 | -34.12 | 26.42 | 0 | 0 | 2 | 0 | 0 | 2 |
| Deer Park | 18.42 | -34.01 | 27.53 | 2 | 0 | 2 | 2 | 2 | 8 |
| The Glen | 18.39 | -33.94 | 28.54 | 0 | 0 | 0 | 0 | 1 | 1 |
| Newlands 2-1 | 18.44 | -33.97 | 28.59 | 2 | 0 | 0 | 0 | 0 | 2 |
| Newlands 3 | 18.44 | -33.98 | 30.28 | 0 | 0 | 2 | 0 | 0 | 2 |
| Newlands 4-2 | 18.45 | -33.97 | 31.34 | 0 | 3 | 1 | 2 | 0 | 6 |
| Tokai Picnic | 18.42 | -34.06 | 31.67 | 1 | 2 | 3 | 0 | 0 | 6 |
| Gemini (De Hel 2) | 18.42 | -34.02 | 32.23 | 0 | 0 | 1 | 0 | 0 | 1 |
| UCT | 18.46 | -33.96 | 33.51 | 0 | 2 | 1 | 2 | 1 | 6 |
| Westlake Golf Course | 18.45 | -34.08 | 34.89 | 0 | 0 | 2 | 3 | 2 | 7 |
| Bishops Court East | 18.39 | -34.10 | 35.28 | 0 | 3 | 3 | 0 | 3 | 9 |
| Newlands Picnic | 18.45 | -33.96 | 36.79 | 2 | 2 | 2 | 2 | 0 | 8 |
| Tierboskloof | 18.37 | -34.04 | 38.19 | 0 | 3 | 2 | 0 | 0 | 5 |
| Sunnycroft | 18.39 | -34.13 | 39.40 | 0 | 0 | 2 | 2 | 0 | 4 |
| Klein Leeukoppie | 18.35 | -34.02 | 41.36 | 0 | 2 | 1 | 0 | 2 | 5 |
| Tamboerskloof 2 | 18.40 | -33.94 | 42.01 | 0 | 0 | 1 | 2 | 1 | 4 |
| Spilhaus | 18.44 | -34.00 | 42.12 | 2 | 1 | 1 | 2 | 1 | 7 |
| Marlene's nest | 18.43 | -34.01 | 42.18 | 2 | 1 | 2 | 2 | 1 | 8 |
| Lower Tokai Forest | 18.43 | -34.06 | 42.22 | 0 | 3 | 2 | 2 | 3 | 10 |
| Pagasvlei | 18.43 | -34.03 | 42.79 | 1 | 0 | 3 | 0 | 1 | 5 |
| Alphen Trail | 18.44 | -34.01 | 45.87 | 0 | 0 | 2 | 2 | 0 | 4 |
| Oakley Avenue | 18.44 | -34.07 | 51.43 | 0 | 1 | 2 | 1 | 3 | 7 |
| Glen Dirk Estate | 18.45 | -34.00 | 53.17 | 0 | 0 | 0 | 4 | 0 | 4 |
| Clovelly Golf Course | 18.45 | -34.00 | 57.54 | 1 | 0 | 0 | 3 | 2 | 6 |
| Ottery 1-2 | 18.50 | -34.01 | 63.32 | 1 | 0 | 1 | 0 | 0 | 2 |
| Sonnehof | 18.44 | -34.04 | 65.78 | 0 | 1 | 2 | 1 | 1 | 5 |
| Alphen Common | 18.45 | -34.02 | 67.93 | 3 | 3 | 1 | 2 | 0 | 9 |
| Audrey's Nest | 18.51 | -33.96 | 68.31 | 0 | 0 | 3 | 2 | 0 | 5 |
| Zonnestraal 2 | 18.46 | -34.01 | 71.15 | 0 | 2 | 1 | 0 | 0 | 3 |
| Fernwood | 18.46 | -34.02 | 73.13 | 2 | 0 | 2 | 0 | 0 | 4 |
| Kliproad Cementary | 18.49 | -34.04 | 75.60 | 0 | 0 | 2 | 1 | 0 | 3 |
| Sangrove | 18.49 | -33.97 | 80.25 | 0 | 2 | 1 | 0 | 0 | 3 |
| Titus Way | 18.46 | -34.03 | 80.56 | 1 | 2 | 2 | 0 | 0 | 5 |

**Table S2**: Matrix showing correlation coefficients of computed Pearson’s correlation between response and predictor variables considered in this study on immune function and antioxidant capacity in urban nestlings of Black Sparrowhawks. Significant correlations are highlighted in bold. Non-enzymatic antioxidant capacity (tAOX), total glutathione (tGSH). Body mass index is calculated as the residual of body mass against tarsus length corrected for sex.

|  | **Haemolysis** | **Haemagglutination** | **Haptoglobin** | **Bactericidal capacity** | **tAOX** | **tGSH** | **Breeding date** | **Rainfall** | **Urban cover** | **Body mass** | **Tarsus length** | **Body mass index** | **Nestling age** |
| --- | --- | --- | --- | --- | --- | --- | --- | --- | --- | --- | --- | --- | --- |
| **Haemolysis** |  | **0.22** | 0.1 | **0.42** | **0.22** | 0.04 | 0.09 | 0.08 | **0.17** | 0.11 | 0.1 | 0.03 | **0.13** |
| **Haemagglutination** |  |  | -0.02 | 0.04 | **-0.27** | **0.15** | 0.02 | -0.01 | -0.05 | 0.11 | 0.1 | 0.02 | 0.04 |
| **Haptoglobin** |  |  |  | 0.2 | **0.24** | -0.06 | -0.12 | 0.07 | -0.07 | **0.16** | 0.07 | **0.13** | 0.03 |
| **Bactericidal capacity** |  |  |  |  | **0.18** | 0.01 | 0.04 | 0.09 | 0.09 | **0.28** | **0.21** | 0.09 | **0.24** |
| **tAOX** |  |  |  |  |  | -0.08 | -0.03 | 0.06 | -0.07 | -0.01 | -0.09 | **0.14** | 0.04 |
| **tGSH** |  |  |  |  |  |  | 0.08 | -0.02 | -0.01 | **0.25** | **0.26** | -0.06 | 0.11 |
| **Breeding date** |  |  |  |  |  |  |  | **-0.57** | **-0.13** | 0.03 | **0.14** | **-0.19** | -0.07 |
| **Rainfall** |  |  |  |  |  |  |  |  | 0.11 | 0.08 | 0.03 | 0.08 | 0.06 |
| **Urban cover** |  |  |  |  |  |  |  |  |  | -0.07 | -0.02 | -0.09 | 0.06 |
| **Body mass** |  |  |  |  |  |  |  |  |  |  | **0.85** | **0.2** | **0.25** |
| **Tarsus length** |  |  |  |  |  |  |  |  |  |  |  | **-0.33** | **0.25** |
| **Body mass index** |  |  |  |  |  |  |  |  |  |  |  |  | 0.02 |
| **Chick age** |  |  |  |  |  |  |  |  |  |  |  |  |  |
| *Computed correlation used pearson-method with listwise-deletion.* | | | | | | | | | | | | | |

**Table S3:** Matrix showing correlation coefficients of computed Pearson’s correlation between response and predictor variables considered for the effect of urban cover on immune response in nestling Black Sparrowhawk. Significant correlations are highlighted in bold. Body mass index is calculated as the residual of body mass against tarsus length corrected for sex.

|  | ***Delta Haptoglobin concentration*** | ***Haptoglobin post challenge*** | ***Haptoglobin pre challenge*** | ***Breeding date*** | ***Rainfall*** | ***Urban cover*** | ***Body mass*** | ***Tarsus length*** | ***Body mass index*** | ***Chick age*** |  |
| --- | --- | --- | --- | --- | --- | --- | --- | --- | --- | --- | --- |
| ***Delta Haptoglobin***  ***concentration*** | | **0.46** | **-0.29** | -0.11 | 0.02 | -0.1 | 0.07 | 0.06 | 0.02 | **0.15** |  |
| ***Haptoglobin post challenge*** |  |  | **0.72** | -**0.18** | **0.21** | -0.12 | **0.26** | **0.24** | 0.05 | **0.26** |  |
| ***Haptoglobin pre challenge*** |  |  |  | -0.11 | **0.21** | -0.05 | **0.22** | **0.22** | 0.03 | **0.16** |  |
| ***Breeding date*** |  |  |  |  | **-0.56** | 0.09 | -0.05 | **0.17** | **-0.29** | 0.03 |  |
| ***Rainfall*** |  |  |  |  |  | -0.07 | 0.07 | -0.04 | 0.13 | 0.04 |  |
| ***Urban cover 2000*** |  |  |  |  |  |  | -**0.20** | -0.03 | **-0.27** | **-0.16** |  |
| ***Body mass*** |  |  |  |  |  |  |  | **0.80** | **0.41** | **0.48** |  |
| ***Tarsus length*** |  |  |  |  |  |  |  |  | **-0.21** | **0.31** |  |
| ***Body mass index*** |  |  |  |  |  |  |  |  |  | **0.35** |  |
| ***Chick age*** |  |  |  |  |  |  |  |  |  |  |  |
| *Computed correlation used pearson-method with listwise-deletion.* | | | | | | | | | | | |
